# Supplementary material for: The Role of the Oral Microbiota in the Etiopathogenesis of Oral Squamous Cell Carcinoma
Source: Microorganisms. 2021 Jul 21;9(8):1549. doi: 10.3390/microorganisms9081549 (PMC8400438; doi:10.3390/microorganisms9081549)
Supplement: Supplementary file 1 [file microorganisms-09-01549-s001.zip › microorganisms-1302526-supplementary.pdf]

microorganisms

MDPI

Table S1. Summary of bacteria that occur in patients with oral squamous cell carcinoma (OSCC). Comparison of specific bacteria´s abundance between case and control/case samples. All specimens were human.

Genus/Species	Abundance in OSCC case samples relative to control/case samples	Case samples from OSCC patients	Control/case samples	Number of participants	Reference
Actinobacillus	Decreased	Saliva	Saliva	3 OSCC patients, 2 individuals without malignity	[39]
Actinomyces	Decreased	Oral mucosa swabs	Oral mucosa swabs	22 cancer patients, 8 pre-cancer patients, 6 healthy individuals	[40]
	Decreased	Oral mucosa swabs	Oral mucosa swabs	40 OSCC patients, 40 controls	[41]
	Decreased	Oral rinsing	Oral rinsing	197 OSCC patients, 51 healthy individuals	[42]
Actinomyces odontolyticus	Decreased	Deep and superficial OSCC tissue	Normal oral mucosa tissue	20 OSCC patients	[13]
Alloprevotella	Increased	Oral mucosa swabs	Oral mucosa swabs	40 OSCC patients, 40 controls	[41]
Campylobacter	Increased	Fresh OSCC tissue	Oral mucosa (deep-epithelium) swabs	20 OSCC patients, 20 healthy individuals	[43]
	Increased	Oral mucosa swabs	Oral mucosa swabs	40 OSCC patients, 40 controls	[41]
Capnocytophaga	Increased	Oral mucosa swabs	Oral mucosa swabs	40 OSCC patients, 40 controls	[41]
	Decreased	Saliva from OSCC patients	Saliva	3 OSCC patients, 2 individuals without malignity	[39]
Capnocytophaga gingivalis	Increased	Saliva from OSCC patients	Saliva	229 OSCC-free individuals, 45 OSCC patients	[14]
Catonella	Increased	Oral mucosa swabs	Oral mucosa swabs	40 OSCC patients, 40 controls	[41]
Cloacibacillus	Increased	Saliva	Saliva	125 OSCC patients, 124 patients with epithelial precursor lesions, 127 healthy individuals OSCC patients	[44]
Dialister	Increased	Oral mucosa swabs	Oral mucosa swabs	40 OSCC patients, 40 controls	[41]
Exiguobacterium oxidotolerans	Increased	Deep and superficial OSCC tissue	Normal oral mucosa tissue	20 OSCC patients	[13]
Eikenella	Increased	Oral mucosa swabs	Oral mucosa swabs from healthy individuals	40 OSCC patients, 40 controls	[41]
Filifactor	Increased	Oral mucosa swabs	Oral mucosa swabs from healthy individuals	40 OSCC patients, 40 controls	[41]
**Genus/Species**	**Abundance in OSCC case samples relative to control/case samples**	**Case samples from OSCC patients**	**Control/case samples**	**Number of participants**	**Reference**

**Genus/Species**	**Abundance in OSCC case samples relative to control/case samples**	**Case samples from OSCC patients**	**Control/case samples**	**Number of participants**	**Reference**
Filifactor alocis	Increased	Oral rinsing	Oral rinsing from healthy individuals	197 OSCC patients, 51 healthy individuals	[42]
Fusobacterium naviforme	Increased	Deep and superficial OSCC tissue	Normal oral mucosa tissue	20 OSCC patients	[13]
	Increased	OSCC tissue	Normal oral mucosa tissue	10 OSCC patiens	[45]
Fusobacterium nucleatum	Increased	Fresh OSCC tissue	Oral mucosa (deep-epithelium) swabs from healthy individuals	20 OSCC patients, 20 healthy individuals	[43]
	Increased	OSCC tissue	Normal oral mucosa tissue	61 OSCC patients, 30 healthy individuals	[46]
	Increased	Saliva	Saliva	4 OSCC pacients (HPV negative), 7 OSCC patients (HPV positive), 6 OSCC patients (HPV negative), 25 healthy individuals	[47]
Fusobacterium periodonticum	Increased	Oral rinsing	Oral rinsing	197 OSCC patients, 51 healthy individuals	[42]
Gemella	Increased	Saliva	Saliva	3 OSCC patients, 2 individuals without malignity	[39]
Gemmiger	Increased	Saliva	Saliva	125 OSCC patients, 124 patients with epithelial precursor lesions, 127 healthy individuals OSCC patients	[44]
Granulicatella	Decreased	Oral mucosa swabs	Oral mucosa swabs	40 OSCC patients, 40 controls	[41]
Haemophilus influenza	Increased	Oral rinsing	Oral rinsing	197 OSCC patients, 51 healthy individuals	[42]
Haemophilus parainfluenzae	Decreased	Fresh OSCC tissue	Oral mucosa (deep-epithelium) swabs	20 OSCC patients, 20 healthy individuals	[43]
	Decreased	Oral rinsing	Oral rinsing	197 OSCC patients, 51 healthy individuals	[42]
Lactobacillus	Increased	Saliva	Saliva	3 OSCC patients, 2 individuals without malignity	[39]
	Increased	Saliva	Saliva	4 OSCC pacients (HPV negative), 7 OSCC patients (HPV positive), 6 OSCC patients (HPV negative), 25 healthy individuals	[47]
Leptotrichia buccalis	Increased	Saliva	Saliva	125 OSCC patients, 124 patients with epithelial precursor lesions, 127 healthy individuals OSCC patients	[44]
	Decreased	Saliva	Saliva	45 OSCC patients, 229 OSCC-free individuals	[14]

Abbreviations: FFPE, formalin-fixed paraffin-embedded

Megasphaera	Decreased	Oral mucosa swabs	Oral mucosa swabs	40 OSCC patients, 40 controls	[41]
Micrococcus luteus	Increased	Deep and superficial OSCC tissue	Normal oral mucosa tissue	20 OSCC patients	[13]
Mycoplasma	Increased	Oral mucosa swabs	Oral mucosa swabs	40 OSCC patients, 40 controls	[41]
Oribacterium	Decreased	Saliva	Saliva	3 OSCC patients, 2 individuals without malignity	[39]
Oscillospira	Increased	Saliva	Saliva	125 OSCC patients, 124 patients with epithelial precursor lesions, 127 healthy individuals OSCC patients	[44]
Parvimonas	Increased	Oral mucosa swabs	Oral mucosa swabs	40 OSCC patients, 40 controls	[41]
	Increased	Saliva	Saliva	4 OSCC pacients (HPV negative), 7 OSCC patients (HPV positive), 6 OSCC patients (HPV negative), 25 healthy individuals	[47]
	Increased	Saliva	Saliva	125 OSCC patients, 124 patients with epithelial precursor lesions, 127 healthy individuals OSCC patients	[44]
Parvimonas micra	Increased	Oral rinsing	Oral rinsing	197 OSCC patients, 51 healthy individuals	[42]
Peptococcus	Increased	Oral mucosa swabs	Oral mucosa swabs	40 OSCC patients, 40 controls	[41]
Porphyromonas gingivalis	Increased	FFPE gingival carcinoma tissue	FFPE non-neoplastic gingival tissue	10 OSCC patients, 5 controls	[12]
	Increased	OSCC tissue	Normal oral mucosa tissue	61 OSCC patients, 30 healthy individuals	[46]
	Increased	Saliva	Saliva	45 OSCC patients, 229 OSCC-free individuals	[14]
Porphyromonas pasteri	Decreased	Oral rinsing	Oral rinsing	197 OSCC patients, 51 healthy individuals	[42]
Prevotella melaninogenica	Increased	Saliva	Saliva	45 OSCC patients, 229 OSCC-free individuals	[14]
	Increased	Deep and superficial OSCC tissue	Normal oral mucosa tissue	20 OSCC patients	[13]
**Genus/Species**	**Abundance in OSCC case samples relative to control/case samples**	**Case samples from OSCC patients**	**Control/case samples**	**Number of participants**	**Reference**
Pseudomonas aeruginosa	Increased	Fresh OSCC tissue	Oral mucosa (deep-epithelium) swabs	20 OSCC patients, 20 healthy individuals	[43]
Roseburia	Increased	Saliva	Saliva	125 OSCC patients, 124 patients with epithelial precursor lesions, 127 healthy individuals OSCC patients	[44]
Rothia	Decreased	Oral mucosa swabs	Oral mucosa swabs	40 OSCC patients, 40 controls	[41]
	Decreased	Oral mucosa swabs	Oral mucosa swabs	22 OSCC patients, 8 pre-cancer patients, 6 healthy individuals	[40]
	Decreased	Oral rinsing	Oral rinsing	21 HPV-negative OSCC patients, 31 HPV-positive OSCC patients	[48]

Rothia mucilaginosa	Decreased	Fresh OSCC tissue	Oral mucosa (deep-epithelium) swabs	20 OSCC patients, 20 healthy individuals	[43]
Selenomonas	Increased	Oral mucosa swabs from OSCC patients	Oral mucosa swabs	40 OSCC patients, 40 controls	[41]
Staphylococcus aureus	Increased	Deep and superficial OSCC tissue	Normal oral mucosa tissue	20 OSCC patients	[13]
Stomatobaculum	Decreased	Oral mucosa swabs	Oral mucosa swabs	40 OSCC patients, 40 controls	[41]
Streptococcus constellatus	Increased	Oral rinsing	Oral rinsing	197 OSCC patients, 51 healthy individuals	[42]
Streptococcus mitis	Increased	Saliva	Saliva	45 OSCC patients, 229 OSCC-free individuals	[14]
	Decreased	Fresh OSCC tissue	Oral mucosa (deep-epithelium) swabs	20 OSCC patients, 20 healthy individuals	[43]
	Decreased	Oral rinsing	Oral rinsing	197 OSCC patients, 51 healthy individuals	[42]
Streptococcus sanguinis	Decreased	OSCC tissue	Normal oral mucosa tissue	61 OSCC patients, 30 healthy individuals	[46]
Treponema	Increased	Oral mucosa swabs from OSCC patients	Oral mucosa swabs	40 OSCC patients, 40 controls	[41]
	Increased	Saliva	Saliva	4 OSCC pacients (HPV negative), 7 OSCC patients (HPV positive), 6 OSCC patients (HPV negative), 25 healthy individuals	[47]
Veillonella	Decreased	Oral mucosa swabs	Oral mucosa swabs	40 OSCC patients, 40 controls	[41]
Veillonella parvula	Decreased	Oral rinsing	Oral rinsing	197 OSCC patients, 51 healthy individuals	[42]
